# Virtual Reality in the Rehabilitation of Patients with Injuries and Diseases of Upper Extremities

**DOI:** 10.3390/healthcare10061124

**Published:** 2022-06-16

**Authors:** Pinar Tokgöz, Susanne Stampa, Dirk Wähnert, Thomas Vordemvenne, Christoph Dockweiler

**Affiliations:** 1 School of Public Health, Bielefeld University, 33615 Bielefeld, Germany; 2 Department of Digital Health Sciences and Biomedicine, School of Life Sciences, University of Siegen, 57068 Siegen, Germany; susanne.stampa@uni-siegen.de (S.S.); christoph.dockweiler@uni-siegen.de (C.D.); 3 Department for Trauma Surgery and Orthopedics, Protestant Hospital of Bethel Foundation, 33617 Bielefeld, Germany; dirk.waehnert@evkb.de (D.W.); thomas.vordemvenne@evkb.de (T.V.)

**Keywords:** rehabilitation, virtual reality, upper extremity, virtual rehabilitation

## Abstract

Upper-extremity injuries and diseases rarely have life-threatening consequences, but failure to manage them properly can result in severe dysfunction. This article presents the current state of using virtual reality to support the rehabilitation process of patients with injuries and diseases of the upper extremities and points out their effects on upper-extremity functions. A scoping review was conducted to provide a comprehensive overview of the field of virtual reality for upper-extremity rehabilitation. PubMed, Web of Science, and the Cochrane Library were searched by two independent researchers between April and May 2021 to identify relevant publications and were examined according to inclusion and exclusion criteria. As a result of the literature review, 11 studies of various target groups were identified. Virtual-reality technologies were categorized into multisensory high-end systems and game-based systems. With respect to functional recovery, technologies based on virtual reality were not inferior to traditional rehabilitation. In addition, the users were highly motivated and satisfied. The results emphasize the need for stronger evidence-based virtual-reality technologies for rehabilitation of injuries and diseases of upper extremities.

## 1. Introduction

Injuries and diseases that concern the apparatus of movement and truss pose a long-term threat to both professional and individual performance, as well as participation in social life. In 2018, diseases of the musculoskeletal system were the most frequent reasons for outpatient rehabilitation among women (74%) and men (67%) in Germany [1]. Most of the complaints affected the upper extremities [2]. Rehabilitation is considered essential for the recovery of patients suffering from injuries and diseases of upper extremities and regaining quality of life.

Rehabilitation is a multifaceted long-term process ranging from inpatient or outpatient rehabilitation up to subsequent rehabilitation services. Accordingly, the conservative treatment includes a multitude of interventions, such as physical therapy, psychological treatment, and activities such as swimming or yoga [3,4]. To maintain the success of rehabilitation, an ongoing execution of acquired changes and a long-term provision of subsequent rehabilitation services are required [5]. With the dissemination of technological innovations, new opportunities arise to redesign rehabilitation services.

A technical innovation highly relevant for rehabilitation is virtual reality (VR). VR is the computer-animated simulation of a three-dimensional environment that can be used in real time [6]. Rehabilitation through VR describes an assistive health technology that is used to recover motor or sensory skills lost due to accident or illness through a virtual but interactive environment [7]. VR consists of a range of technologies that can be used to artificially generate sensory information in the form of a virtual environment that is interactive and perceived as similar to the real world. In addition, audiovisual feedback functions can improve compliance and therapeutic effectiveness [7].

In rehabilitation, VR represents a valid and reliable tool for joint and function [8]. It is a cost-saving alternative and enables personalizing treatment, motivating patients, and increasing their compliance and functional recovery [9]. VR is also generally commercially available and can be used for home-based rehabilitation [10,11]. This may reduce the work burden on professionals, because it requires minimal supervision [12]. An increasing number of VR technologies are supplemented by playful concepts, whereby various elements, dynamics, and mechanics are used. For example, it is possible for virtual environments to be presented on screens or displayed in VR glasses, augmented with simultaneous auditory presentations, closely approximating the complexity of the everyday world [13]. In combination with three-dimensional motion analysis, VR technologies have great potential for the rehabilitation of upper-extremity functions [14]. The design of the systems is often similar; one or more forms of sensor technology record the user’s movements, which are presented in a playful and everyday manner [15]. Thorough system design, the patients should be able to implement the idea of movement specification. At the same time, it is possible for professionals to change the structure and severity of training [14].

The effectiveness of VR in neurorehabilitation has been studied extensively in individuals with cerebral palsy [16] and especially stroke [15,17]. In spite of VR´s promising effects for rehabilitation, it lacks routine use in practice. Moreover, its effectiveness for the rehabilitation of upper extremities beyond neurological disorders is insufficiently explored. Patients with neurological diseases might suffer from upper-extremity dysfunction, but there might be differences in treatment goals, which require consideration. Therefore, the aim of this scoping review was to present the current state of VR technologies being used in the rehabilitation of upper-extremity injuries and diseases other than neurological disorders and to examine their impact on functional recovery.

## 2. Materials and Methods

This scoping review was conducted using the framework of Arksey and O’Malley [18], described in detail by Levac et al. [19]. The databases PubMed, Web of Science, and the Cochrane Library were searched between April and May 2021. The search was updated in January 2022. Using relevant search terms and Medical Subject Headings (MeSH) such as “rehabilitation”, “virtual reality”, and “upper-extremity diseases”, a search syntax was developed.

Two independent reviewers judged the eligibility of retrieved studies by title and abstract, as well as according to inclusion and exclusion criteria. Duplicates were sorted out, and appropriate studies were included in a second screening. Thus, in the next step, the studies were assessed on the basis of full texts and with renewed consideration of inclusion and exclusion criteria. Studies that were not published in English or German, were available as abstracts only, or focused on neurological diseases solely were excluded. Studies that used rehabilitation approaches based on VR as a training tool, reported at least one outcome related to functional recovery, and were published in English or German between the years 2011 and 2021 were included. If disagreement existed, a consensus was reached through discussion.

A standardized data extraction form was used to collect information related to the following aspects:

- Authors,
- Country,
- Study design,
- Sample size,
- Study population,
- Intervention characteristics,
- Relevant outcomes and results.

These data were used for precise planning and preparation of the qualitative synthesis.

## 3. Results

**Figure 1:**
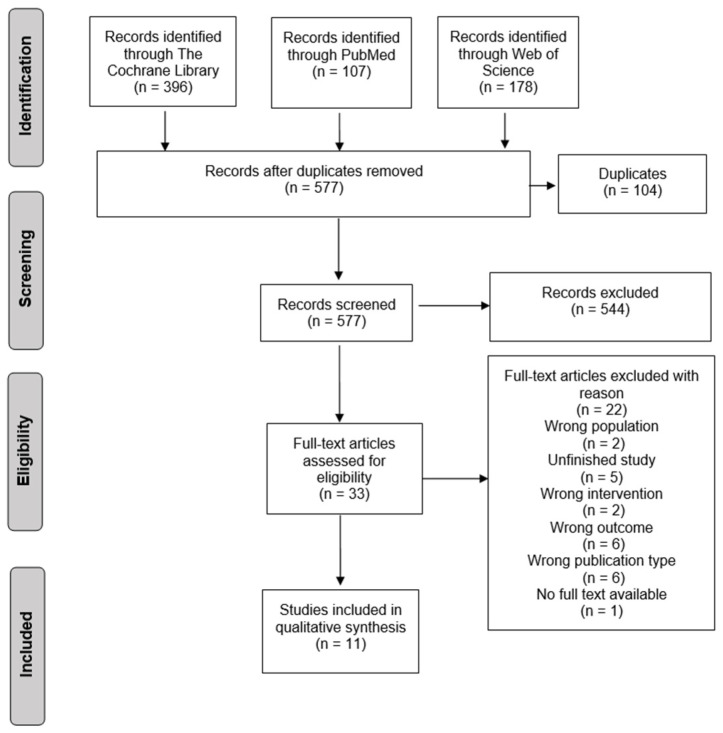
Flow diagram of literature search and selection process.

The initial search procedure retrieved 681 articles. After duplicate removal and screening, 11 studies were considered eligible for inclusion (see Figure 1).

### 3.1. Study Characteristics

Of the included studies, five studies were conducted as pilot randomized controlled trials [20,21,22,23,24]. Three studies each were designed as pilot studies [25,26,27] and randomized controlled trials [28,29,30]. Sample size ranged from six to 57 participants when intervention and control groups were summed up. There was variability with regard to study population. Four studies considered functional disorders after burns of the upper extremities [21,22,23,30], while two studies considered functional disorders of the upper extremities after surgical treatment of breast cancer [26,28]. Further indications were chronic pain syndrome of upper extremity [25], shoulder injuries [20], impingement syndrome [29], frozen shoulder [27], and rheumatoid arthritis [24].

Consequently, there was heterogeneity in the parameters measured and assessment methods used in the studies, with a predominant focus on pain perception and dysfunction of the upper extremity. The most common evaluation methodology used was the visual analog scale (VAS) to assess pain and range of motion (ROM) to assess upper-extremity function.

The VR technologies described in the studies can be divided into two categories. The first constituted multisensory high-end systems that enable complete immersion. In the pilot study of Chau et al. (2020), participants used the HTC Vive VR system, which consisted of a wired headset and two handheld motion controllers. Participants were approved for up to 10 sessions of VR therapy, with each session lasting approximately 45 min to 1 h. The sessions consisted of guided visualization exercises and interactions with the virtual environment. Activities included, for example, washing hands, sorting dishware, or arranging utensils [25]. The BrightArm Duo Rehabilitation System in the pilot study of House et al. (2016) is an integrative virtual rehabilitation game system consisting of different exercises for motor, emotive, and cognitive function. For instance, participants were asked to use paddle avatars to bounce a virtual ball toward an array of crates. The duration of the VR therapy sessions progressed from 20 to 50 min of training over a period of 8 weeks, with two sessions every week. All games had progressively higher difficulty levels over the study duration, which were combined with higher gravity loading and longer training sessions to keep the participants challenged [26]. In the study of Lee et al. (2016), an interactive motor training VR system was designed to formulate a goal-directed shoulder rehabilitation, which assisted participants with motor rehabilitation. Sensors were secured to the shoulder joints to measure and record ROM, while participants underwent various exercises consisting of shoulder ROM exercises, shoulder muscle strengthening, and core muscle strengthening. The exercise sessions were 40 min twice a week for 4 weeks. The practice time for each exercise was based on the participants’ progress and was determined by the therapists [27]. In the study of Joo et al. (2020), the VR rehabilitation tool RAPAEL Smart Glove was designed for the distal upper extremities. All participants received a 4 week intervention, consisting of 20 sessions for 60 min per day. Participants of the intervention group received 30 min of standard therapy and 30 min of exercises with the VR system. Exercises of the VR system demanded volitional movements such as forearm pronation/supination or wrist flexion/extension. Visual and audio feedback informed participants of success or failure. Participants of the control group received 60 min standard therapy, which comprised ROM exercises or strengthening exercises. The exercises in both groups were comparable and adapted to each participants’ performance. The intervention frequency and duration did not differ between both groups. Three therapists who had experience with VR systems and conventional therapy supervised the study [30].

On the other hand, there are commercially available game-based systems, such as Nintendo Wii and Xbox Kinect, which are designed to be lower-threshold and less expensive. The majority of the studies included in this work (*N* = 7) used game-based systems for upper-extremity rehabilitation [20,21,22,23,24,28,29].

Participants in the study of Dahl-Popolizio et al. (2014) performed a protocol of active, active assistivem and passive ROM exercises for six sessions using Microsoft Kinect, where the movements attempted to match the movements of an avatar, which was displayed on a screen. Participants in the control group received similar exercises for six sessions but without Microsoft Kinect and with minimal therapeutically support [20]. In the study of Voon et al. (2016), all participants were requested to attend the study for 1 week and to perform two 30 min exercise sessions daily. Participants in the intervention group were asked, for each session, to perform 15 min of routine physiotherapy exercises followed by a minimum of 15 min of Xbox Kinect game play, consisting of exercises such as darts or bowling. Participants in the control group performed, for each session, a minimum of 30 min of the same routine physiotherapy regime [23]. Similarly, in the study of Feyzioglu et al. (2020), the participants of the intervention group received Xbox Kinect therapy, while the control group received standardized routine physiotherapy. Both groups received the therapy for 45 min per session and two times a week for 6 weeks. Additionally, all participants were given the same home exercise program except for the session days [28].

In the study of Yohannan et al. (2012), all participants received three consecutive sessions of traditional passive ROM and joint-specific exercises for 15 min. This was followed by an additional 15 min of Nintendo Wii exercises consisting of games such as wall finger climbs, overhead ball toss, or bouncing a physioball for those in the intervention group or 15 min of a therapist-chosen exercise for those in the control group. In the control group, although therapy was tailored by interventional therapists, the movements were comparable with the intervention group. Interventional therapists were guided by scripts to provide standardized therapy [21]. The study of Parker et al. (2016) lasted a maximum of 7 days. During this time, in addition to their individualized routine exercises, participants of the intervention group completed up to five individual days of twice-daily exercise with Nintendo Wii, whereby each session lasted 20–30 min. The sessions were self-directed and consisted of an individual playing specific games with a standardized order according to the injury and the limb involved. Participants in the control group received routine individualized exercises that were comparable with the intervention group [22]. In the cross-sectional study of Zernicke et al. (2016), participants of the intervention group started with exercises using Nintendo Wii, while participants in the control group completed conventional physical exercises for 12 weeks. Afterward, participants were crossed over for another period of 12 weeks. Participants in the intervention group could select from at least 46 exercises of different fields such as aerobic, muscle strengthening, or balance. Exercises in the control group were designed by physiotherapists and based on strength training, coordination, joint mobility, and relaxation. According to the training schedule, each participant was stimulated to exercise three times a week for approximately 30 min per session [24]. Participants in the study from Pekyavas and Ergun (2017) were included in the intervention group receiving supervised exercises with Nintendo Wii for 6 weeks, two days per week, and 45 min for day. The sessions consisted of warming and cooling periods and several training games comprising boxing, bowling or tennis games accompanied by an avatar. Participants in the control group received a home exercise program for 6 weeks, two days per week, and 45 min per day. Exercises were similar to the intervention group and consisted of resistive and non-resistive exercises [29].

In two studies, participants using the VR technology received continuous support from trained professionals [21,26]. In another two studies, participants received audiovisual feedback on movement execution [20,30].

### 3.2. Effectiveness of VR Technologies

The results of the pilot studies in pre–post design showed significant improvements with regard to pain and upper-extremity function [26,27]. The study by Chau et al. did not find any statistically significant improvements in the parameters measured [25].

Varying results were obtained in the randomized controlled trials. Rarely, the VR technologies in the included studies were associated with measurable benefits related to pain perception and upper-extremity function. For example, the study results of Parker et al. showed significant improvements in pain (*p* = 0.0019) favoring the intervention group using the VR technology, while no statistically significant differences were detectable in range of motion [22]. In contrast, the intervention group in the study by Joo et al. was associated with statistically significant improvements in the subscales “picking up small objects” (*p* < 0.001) and “pain” (*p* = 0.002) of the Jebsen–Taylor hand function test (JTT) [30]. Pekyavas and Ergun also obtained similar results, where, in terms of single parameters of functionality and pain perception, the intervention group using the VR technology was superior to traditional rehabilitation, while, in other parameters, no statistically significant differences were detectable [29]. Four studies found no statistically significant differences at all with regard to upper-extremity function or pain between participants using the VR technology and participants receiving traditional rehabilitation [20,21,23,24].

Beyond that, results related to user satisfaction and motivation were mostly consistent. Overall, users were satisfied with the interventions used [20,23,30] and motivated to complete training sessions [24,25,26]. All analyzed studies are summarized in Table 1.

## 4. Discussion

Numerous reviews [15,17,31] have already shown the successful use of VR technologies for rehabilitation, especially in the field of neurological disorders. For rehabilitation of musculoskeletal diseases, in contrast, only a few publications can be found [32,33]. Therefore, this scoping review aimed to provide an overview of VR technologies used for rehabilitation of upper-extremity injuries and diseases beyond neurological impairments.

Existing evidence of VR technology effectiveness in patients with upper-extremity injuries and diseases is inconclusive yet promising. Findings from this scoping review indicate that VR-based interventions are not inferior to traditional rehabilitation and might have an added advantage for functional recovery and pain reduction. Future studies of high quality are necessary to reach a more solid conclusion.

The number of studies that used game-based VR technologies is remarkable. The integration of low-cost game-based VR technologies can allow healthcare specialists to precisely create, deliver, and control complex and dynamic environments for user interaction. Although such systems were not explicitly developed for rehabilitation, they might be motivating and cost-effective alternatives. As such, game-based VR rehabilitation exercises that are easily adapted to individual user needs will become a valuable adjunct to conventional therapy in inpatient, outpatient, and home-based care settings. However, limited options for customizing and adapting game-based contents to the needs of particular injuries or diseases could also be a disadvantage. Furthermore, visualization of content includes many different elements and stimuli, which might be overwhelming. Additionally, movements to be performed are often too nonspecific and incompatible with therapeutic goals [34].

Future research directions should consider the potential of VR-based systems to increase the efficiency of training in terms of human resources [35]. With VR, therapy sessions can be automated and, therefore, can be completed without the constant supervision of a therapist. Furthermore, a VR system can even be designed for a patient’s home, removing the burden of clinical visits. While the vision for home-based rehabilitation is compelling for economic and technical reasons, professional and user-centered issues will also need to be considered concomitantly as the technology evolves [36]. Thus, the appropriate development and use of the VR systems must always be governed by evidence-based guidelines. Indeed, this is also important for user perspectives [37]. Several studies addressed the challenges of VR in a general or specific field from the user’s perspective [38,39]. In this context, three categories of technical, practical, and user-based challenges for implementing and using a VR-based system have been discussed [40]. One main limitation includes the requirement for specialist technical skills [41]. It is necessary to educate and train clinical experts, as well as patients, in the proper and professional use of VR systems as useful tools for rehabilitation. The method of training users for the necessary skills should be performed considering their age, level of technical literacy, and previous experience with VR systems [42]. It is obvious to educate specialists to gain an important impact on the more effective use of VR.

Most of the VR technologies used in the studies included motion detection systems, in which the virtual environment is presented on large screens. Thus, the user sees the simulated environment and can interact with it. An advantage of this is that the system might encourage users to perform movements naturally. Consequently, VR-based rehabilitation is an effective way for people with upper-extremity injuries and diseases to cope with their motor impairment and regain the ability of performing activities of daily living (ADL) and self-care, which refer to the activities carried out to live an independent life, e.g., grooming or preparing food [43]. Examples that leverage VR for the rehabilitation of ADL include the use of a virtual kitchen for training meal preparation task or the use of a virtual supermarket for practicing shopping tasks [44] for people suffering from traumatic brain injury [45]. Another example is the training of manual skills with an exoskeleton robot also equipped with an actuator to assist shoulder movement. ADL tasks such as cooking, cleaning, and using a ticket machine were trained using the VR system, where the assist-as-needed strategy was adopted to provide guiding force when necessary [46]. Additionally, for successful rehabilitation, motivation of patients is a driving factor [6]. By embedding repetitive exercises in a playful and everyday manner, through virtual environments, motivation and adherence might be achieved [9]. This provides the opportunity to train in real-world scenarios but in a risk-free, graded fashion. The majority of the studies included in this review consisted of up to 10 training sessions and approximately 30 min of exercise time per session, whereby the difficulty and intensity of exercises were adaptable to individuals’ performance. This is important to consider when designing VR exercises since the Yerkes–Dodson law, first explained by Yerkes and Dodson in 1908, describes a relationship between arousal or motivation and performance. It indicates that a low level of task difficulties elicits linear responses. Reaching a higher level of difficulty, the relationship becomes inverse, and increases in arousal or motivation could cause a decrease in performance [47]. An intensive training could reach a point when a higher intensity is necessary to push the functional improvements and patient motivation further, e.g., longer session duration or more sessions per week [48]. Furthermore, the use of VR allows the user’s movement to be quantified and saved for analysis and tracking of performance. Specialists can benefit from the recording of performance data to analyze quality of movement and track patient progress within and across sections. Users may benefit from being able to view visualizations of their activity from different perspectives. Additionally, specific and immediate feedback on movement performance might also have a positive effect on motivation and adherence.

It is important to note a few limitations. First, only articles in the English and German language were included. Furthermore, studies that assessed neurological disorders were excluded. Because of this, only 11 studies could be identified, which again highlights that the potential of VR technologies for the rehabilitation of upper-extremity diseases and injuries is still underexplored. The heterogeneity of endpoints and assessment tools, as well as the small sample sizes in the studies, limits the comparability and interpretation of the results. Additionally, it was not possible to obtain conceptual or concrete content-related details of VR technologies.

## 5. Conclusions

In conclusion, this scoping review showed that especially game-based VR systems are highly prevalent in the context of rehabilitation and seem to be gaining importance to enhance motivation and support therapy. As presented, the literature to date strongly suggests that these technologies are poised to have a major impact on evaluation and intervention for motor and functional rehabilitation because of the unique attributes of VR-based therapy. These attributes make it highly suitable for the achievement of many rehabilitation goals, including the encouragement of active learning, the provision of challenging but safe environments, the flexibility of individualized and graded treatments, the power to motivate patients to perform to their utmost ability, and the capacity to record objective measures of performance.

VR systems are being directed at the rehabilitation of motor deficits to help provide recreational opportunities for people with upper-extremity dysfunction. VR-mediated rehabilitation has yielded significant improvements in upper-extremity recovery, especially regarding range of motion and pain reduction. VR systems show promise for training in activities of daily living, including use of a virtual kitchen, street-crossing, and wayfinding environment. Evidence from this scoping review suggests that VR technologies have the potential to become an effective tool in the rehabilitation of upper-extremity functions. Although the results do not indicate VR systems to be significantly more beneficial than routine physiotherapy, VR systems are comparable with existing traditional rehabilitation procedures and can be used as an alternative or adjunct for the rehabilitation of upper-extremity injuries and diseases. Advantages are mainly seen in the increased motivation to perform therapy tasks and the simulated and risk-free training of functional exercises with a stronger intensity than traditional rehabilitation.

Overall, VR offers a unique medium in which rehabilitation can be offered within a functional, purposeful, and motivation context, which can be readily graded and documented. Especially game-based VR technologies are becoming more accessible and cost-effective; however, they are not yet fully provided in regular care. The successful and extensive implementation of VR technologies in rehabilitation might be possible when the technologies are easily integrated in the everyday life of patients and professionals. For this purpose, it will be necessary to design and evaluate VR technologies in a participatory and user-oriented way. It remains exciting to learn about the effects that future developments will report, including user-specific perspectives.

## Figures and Tables

**Table 1:** Characteristics of included studies.

Study	Country	Study Design	Sample Size	Study Population	Intervention Characteristics	Outcomes	Results
Dahl-Popolizio et al. [20]	USA	Randomized controlled pilot study	IG: 4 CG: 4	Shoulder injuries	IG: Microsoft Kinect motion tracking technology. Participant movement was displayed on a large screen, which was mounted to a motion sensor and could detect in real time. Participants received audiovisual feedback. CG: Standard rehabilitation program.	Shoulder functionality assessed with ROM and FOTO; pain assessed with VAS; satisfaction assessed with a 10-point Likert scale	All measured variables were not significantly different between the two groups. Participants of IG reported high satisfaction with VR system.
Yohannan et al. [21]	USA	Randomized controlled pilot study	IG: 11 CG: 12	Burns	IG: Nintendo Wii fit/sports. Participants first received a standard rehabilitation program for 15 min and then VR exercises mounted to a motion sensor. Participants were supported by trained professionals. CG: Standard rehabilitation program.	Functionality assessed with ROM; pain assessed with VAS	All measured variables were not significantly different between the two groups.
Parker et al. [22]	Australia	Randomized controlled pilot study	IG: 12 CG: 10	Burns	IG: Nintendo Wii fit/sports. Participants first received a standard rehabilitation program and then VR exercises mounted to a motion sensor. CG: Standard rehabilitation program.	Functionality assessed with ROM; pain assessed with VAS	Patients who participated in VR showed significant improvements in pain perception. There were no significant differences in ROM between the two groups.
Voon et al. [23]	Australia	Randomized controlled pilot study	IG: 15 CG: 15	Burns	IG: Xbox 360 Kinect. Participant movement was displayed on a large screen, which was mounted to motion sensor and could detected in real-time, while participants interacted with the virtual environment. Participants first received a standard rehabilitation program for 15 min and then VR exercises for 15 min. CG: Standard rehabilitation program for 30 min.	Functionality assessed with Quick-DASH; satisfaction assessed with VAS; pain assessed with a 10-point Likert scale	Patients who participated in VR showed significant improvements in satisfaction with rehabilitation. There were no significant differences in Quick-DASH and VAS.
Zernicke et al. [24]	Germany	Randomized controlled pilot study	IG: 15 CG: 15	Rheumatoid arthritis	IG: Nintendo Wii fit plus. Participants first received different VR exercises for12 weeks and then 12 weeks of standard rehabilitation. CG: Participants first a received standard rehabilitation program for 12 weeks and then VR exercises for the next 12 weeks.	Functionality assessed with HAQ-DI; pain assessed with VAS; quality of life assessed with SF-36; muscle strength assessed with a dynamometer	All measured variables were not significantly different between the two groups.
Chau et al. [25]	USA	Pilot study	*N* = 8	Chronic pain syndrome	HTC Vive. VR system using wired headset, two handheld motion controllers, and two base stations, which provided boundaries and tracking system of the virtual space. Headset and controllers allowed real-time 3D motion tracking. Exercising with virtual activities such as washing hands, sorting dishware, and arranging utensils.	Pain assessed with VAS, SF-MPQ, and WBF	The were no significant changes in measured variables. Participants tolerated the VR system well and were motivated to continue the rehabilitation program until the end.
House et al. [26]	USA	Pilot study	*N* = 6	Chronic pain syndrome after breast cancer surgery	The BrightArm Duo System is an experimental robotic platform that modulates gravity loading on the upper extremities, consisting of a low-friction robotic rehabilitation table, computerized forearm supports, and a screen that displays motion tracking in real time. Participants were supported by trained professionals	Pain assessed with NRS; functionality assessed with Fugl–Meyer assessment, JTT, and ROM; mobility assessed with Chedokee arm and hand activity inventory-9; activities in daily living assessed with UEFI-20	Significant changes in pain perception and functionality assessed with ROM after 4 weeks and significant changes in daily living assessed with UEFI-20 after 8 weeks were observed.
Lee et al. [27]	China	Pilot study	*N* = 16	Frozen shoulder	Interactive motor training system involving shoulder joint stretching and muscle strengthening. The 3D game engine software was adopted to formulate a goal-directed shoulder rehabilitation program. Sensors were secured to the shoulder joints to record movement execution while patients were undergoing various exercises.	Shoulder flexibility assessed with CMS; functionality assessed with ROM; muscle strength assessed with a dynamometer	Significant changes in the measured variables were recorded in the study period.
Feyzi-oglu et al. [28]	Turkey	Randomized controlled study	IG: 20 CG: 20	Functional impairment of upper extremities after breast cancer surgery	IG: Xbox 360 Kinect. Participant movement was displayed on a large screen, which was mounted to a motion sensor and could detect in real time, while participants interacted with the virtual environment. CG: Standard rehabilitation program.	Functionality assessed with ROM and DASH; muscle strength and flexibility assessed with a dynamometer	Significant changes in measured variables were seen in both groups, while effect sizes in CG were greater than in IG.
Pekyavas & Ergun [29]	Turkey	Randomized controlled study	IG: 15 CG: 15	Impingement syndrome	IG: Nintendo Wii. Participants received differentVR exercises, which were displayed on a large screen in real time. CG: Standard rehabilitation program.	Pain assessed with VAS; clinical symptoms assessed with Neer and Hawkins tests; functionality assessed with LSST, SRT, and SAT; pain and impairments in daily living assessed with SPADI	Significant differences were observed in Neer test but not Hawkins test, favoring IG. Significant differences were observed in SRT, SAT, and SPADI, favoring IG. No significant differences were seen in pain perception assessed with VAS.
Joo et al. [30]	South Korea	Randomized controlled study	IG: 28 CG: 29	Burns	IG: RAPAEL Smart Glove. An exoskeleton type of glove and VR system were used, which could be operated through active movement. The software could be used to visualize the virtual hands in the VR tool according to data gathered by the glove-shaped sensor device. Participants received audiovisual feedback. CG: Standard rehabilitation program.	Functionality assessed by JTT and MHQ; grip strength assessed by grasp and pinch power test	Significant differences in subscales “picking up small objects” and “simulated feeding” of the JTT were recorded, favoring IG. Significant differences in the subscales “daily activity”, “pain”, “work”, and “satisfaction” of the MHQ were recorded, favoring IG. No significant differences were seen in grip strength between both groups.

IG: intervention group, CG: control group, ROM: range of motion, VAS: visual analog scale, FOTO: focus on therapeutic outcome scale, VR: virtual reality, Quick-DASH: disabilities of arm, shoulder, and hand, HAQ-DI: health assessment questionnaire, disability index, SF-36: short-form 36, SF-MPQ: short-form McGill pain questionnaire, WBF: Wong–Baker faces pain rating scale, NRS: numeric rating scale, UEFI-20: Upper-extremity functional index 20, JTT: Jebsen–Taylor hand function test, CMS: constant Murley score, LSST: lateral scapular slide test, SRT: scapular retraction test, SAT: scapular assistance test, SPADI: shoulder pain and disability index, MHQ: Michigan hand questionnaire.

## Data Availability

Not applicable.
